# 85. Improved Survival with Dual Immunomodulator Treatment of ARDS by blocking C5a and IL-6 Activity: Subanalysis from the PANAMO Study in Critically Ill COVID-19 Patients

**DOI:** 10.1093/ofid/ofae631.022

**Published:** 2025-01-29

**Authors:** Roy F Chemaly, Alexander Vlaar, Endry H T Lim, Bruce P Burnett, Camilla Chong, simon Rückinger, Robert Zerbib, Raymond M Panas, Renfeng Guo, Niels Riedemann, Diederik van de Beek

**Affiliations:** University of Texas MD Anderson Cancer Center, Houston, TX; Amsterdam UMC, Amsterdam, Noord-Holland, Netherlands; Amsterdam UMC, Amsterdam, Noord-Holland, Netherlands; InflaRx GmbH, InflaRx Pharmaceuticals, Inc., Fuquay Varina, NC; InflaRx GmbH, London, England, United Kingdom; Metranomia Clinical Research GmbH, Munich, Bayern, Germany; InflaRx GmbH, London, England, United Kingdom; InflaRx Pharmaceuticals, Inc., Pittsburgh, Pennsylvania; InflaRx GmbH, London, England, United Kingdom; InflaRx GmbH, London, England, United Kingdom; Amsterdam UMC, Amsterdam, Noord-Holland, Netherlands

## Abstract

**Background:**

Various pre-clinical and clinical studies suggest an interplay between complement C5a/C5a receptor (R1) and interleukin 6 (IL-6) signaling. C5a boosts IL-6 levels and IL-6 increases C5aR1 in inflammation and sepsis models (Fig 1). Blocking IL-6 signaling, e.g. with tocilizumab (Toci), in patients with acute coronary artery disease and with antibodies in pre-clinical studies in different tissues significantly reduces C5aR1 expression. In addition, inhibiting C5a with vilobelimab (Vilo) in COVID-19 patients with acute respiratory distress syndrome (ARDS) also improves survival as shown in a multicenter, double-blind, randomized, placebo-controlled, phase 3 trial (PANAMO, NCT04333420). COVID-19 patients with ARDS who receive Toci in addition to Vilo may serve as a model for the interplay between IL-6 and the C5a/C5aR1 axis to improve survival in ARDS.

**Figure 1:**
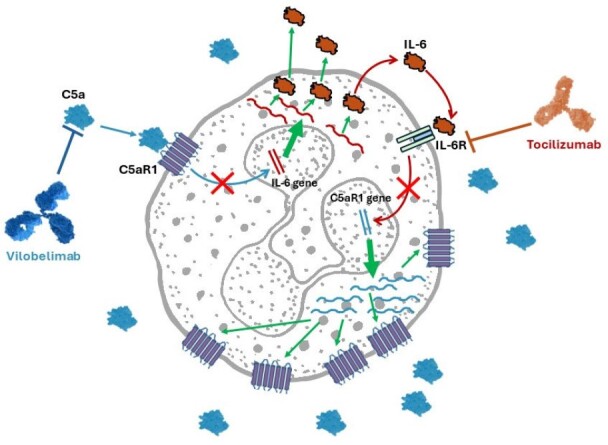
C5a induction of IL-6 and IL-6 induction of C5aR1. Induction of IL-6 is blocked by vilobelimab binding to C5a and tocilizumab blocks IL-6 binding to IL-6 receptor decreasing induction of C5aR1.

**Methods:**

COVID-19 patients with ARDS in the Phase III PANAMO study (N=368) received up to six Vilo 800mg infusions or placebo (Plc) plus corticosteroids and anticoagulants (standard-of-care) within 48 hours of intubation over a 22-day period. A post-hoc Cox regression analysis was performed for 28- and 60-Day all-cause mortality in a subgroup of patients (n=60) who also received prior (within -7 days of Vilo administration) or concomitant Toci. Safety was also assessed.

**Figure 2:**
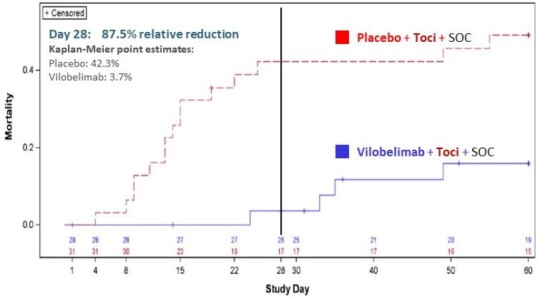
Kaplan Meier Survival Curve of COVID-19 Patients with ARDS Treated with Tocilizumab (Toci) prior (within 7 days) of Vilobelimab+SOC and Placebo+SOC. SOC= Standard-of-Care; corticosteroids and anticoagulants.

**Results:**

Vilo+Toci (n=29) and Plc+Toci (n=31) showed point estimates for 28-Day all-cause mortality of 3.7% and 42.3% (HR 0.073; 95%CI:0.01-0.56, p=0.012), while 60-Day all-cause mortality point estimates were 15.9% and 49.1% (HR 0.245; 95%CI:0.08-0.74, p=0.013), respectively (Fig 2). Related TEAEs were similar in both groups (28.6% *vs* 25.8%). Serious TEAEs occurred in fewer patients receiving Vilo+Toci (42.9%) *vs* Plc+Toci (67.7%). Infections and infestations were slightly lower for Vilo+Toci (53.6%) *vs* Plc+Toci (64.5%).

**Conclusion:**

This post-hoc analysis demonstrated that dual immunomodulator inhibition of the C5a/C5aR1 axis and IL-6 signaling improved survival in a subgroup of COVID-19 patients with ARDS without impacting safety. Dual immunomodulator treatment with vilobelimab and tocilizumab could potentially be used in different ARDS populations to produce a similar effect. Larger and well-controlled studies are warranted to test this hypothesis.

**Disclosures:**

**Roy F. Chemaly, MD/MPH**, AiCuris: Advisor/Consultant|AiCuris: Grant/Research Support|Ansun Pharmaceuticals: Advisor/Consultant|Ansun Pharmaceuticals: Grant/Research Support|Astellas: Advisor/Consultant|Eurofins-Viracor: Grant/Research Support|InflaRX: Advisor/Consultant|Janssen: Advisor/Consultant|Karius: Advisor/Consultant|Karius: Grant/Research Support|Merck/MSD: Advisor/Consultant|Merck/MSD: Grant/Research Support|Moderna: Advisor/Consultant|Oxford Immunotec: Advisor/Consultant|Oxford Immunotec: Grant/Research Support|Roche/Genentech: Advisor/Consultant|Roche/Genentech: Grant/Research Support|Shinogi: Advisor/Consultant|Takeda: Advisor/Consultant|Takeda: Grant/Research Support|Tether: Advisor/Consultant **Alexander Vlaar, MD, PhD**, InflaRx GmbH: Advisor/Consultant **Bruce P. Burnett, PhD**, InflaRx Pharmaceuticals, InflaRx GmbH: Employee **Camilla Chong, MD**, InflaRx GmbH: Employee **simon Rückinger, PhD**, InflaRx GmbH: Advisor/Consultant **Robert Zerbib, MSc**, InflaRx GmbH: Advisor/Consultant **Raymond M. Panas, PhD**, InflaRx Pharmaceuticals, InflaRx GmbH: Employee **Renfeng Guo, MD**, InflaRx GmbH: Board Member|InflaRx GmbH: Chief Scientific Officer|InflaRx GmbH: Ownership Interest|InflaRx GmbH: Stocks/Bonds (Public Company) **Niels Riedemann, MD, PhD**, InflaRx GmbH: Board Member|InflaRx GmbH: Chief Executive Officer|InflaRx GmbH: Ownership Interest|InflaRx GmbH: Stocks/Bonds (Public Company) **Diederik van de Beek, MD, PhD**, InflaRx GmbH: Advisor/Consultant

